# Prioritizing Genomic Drug Targets in Pathogens: Application to Mycobacterium tuberculosis

**DOI:** 10.1371/journal.pcbi.0020061

**Published:** 2006-06-09

**Authors:** Samiul Hasan, Sabine Daugelat, P. S. Srinivasa Rao, Mark Schreiber

**Affiliations:** Novartis Institute for Tropical Diseases (NITD), Chromos, Singapore; Pfizer, United States of America

## Abstract

We have developed a software program that weights and integrates specific properties on the genes in a pathogen so that they may be ranked as drug targets. We applied this software to produce three prioritized drug target lists for *Mycobacterium tuberculosis,* the causative agent of tuberculosis, a disease for which a new drug is desperately needed. Each list is based on an individual criterion. The first list prioritizes metabolic drug targets by the uniqueness of their roles in the M. tuberculosis metabolome (“metabolic chokepoints”) and their similarity to known “druggable” protein classes (i.e., classes whose activity has previously been shown to be modulated by binding a small molecule). The second list prioritizes targets that would specifically impair *M. tuberculosis,* by weighting heavily those that are closely conserved within the Actinobacteria class but lack close homology to the host and gut flora. M. tuberculosis can survive asymptomatically in its host for many years by adapting to a dormant state referred to as “persistence.” The final list aims to prioritize potential targets involved in maintaining persistence in M. tuberculosis. The rankings of current, candidate, and proposed drug targets are highlighted with respect to these lists. Some features were found to be more accurate than others in prioritizing studied targets. It can also be shown that targets can be prioritized by using evolutionary programming to optimize the weights of each desired property. We demonstrate this approach in prioritizing persistence targets.

## Synopsis

The search for drugs to prevent or treat infections remains an urgent focus in infectious disease research. A new software program has been developed by the authors of this article that can be used to rank genes as potential drug targets in pathogens. Traditional prioritization approaches to drug target identification, such as searching the literature and trying to mentally integrate varied criteria, can quickly become overwhelming for the drug discovery researcher. Alternatively, one can computationally integrate different criteria to create a ranking function that can help to identify targets. The authors demonstrate the applicability of this approach on the genome of *Mycobacterium tuberculosis,* the organism that causes tuberculosis (TB), a disease for which new drug treatments are especially needed because of emerging drug-resistant strains. The experiences gained from this work will be useful for both wet-lab and informatics scientists working in infectious disease research; first, it demonstrates that ample public data already exist on the M. tuberculosis genome that can be tuned effectively for prioritizing drug targets. Second, the output from numerous freely available bioinformatics tools can be pushed to achieve these goals. Third, the methodology can easily be extended to other pathogens of interest. Currently studied TB targets are also highlighted in terms of the authors' ranking system, which should be useful for researchers focusing on TB drug discovery.

## Introduction

### The Need for Tools to Rapidly Identify Drug Targets

The cost of research and development in the pharmaceutical industry has been rising steeply and steadily in the last decade, but the amount of time required to bring a new product to market remains around ten to fifteen years [1]. This problem has been labeled an “innovation gap,” and it necessitates investment in inexpensive technologies that shorten the length of time spent in drug discovery.

The target identification stage is the first step in the drug discovery process [2] and as such can provide the foundation for years of dedicated research in the pharmaceutical industry. As with all the other steps in drug discovery, this stage is complicated by the fact that the identified drug target must satisfy a variety of criteria to permit progression to the next stage. Important factors in this context include homology between target and host (to prevent host toxicity such homology must be low or nonexistent [3]); activity of the target in the diseased state [4,5]; and the essentiality of the target to the pathogen's growth and survival [6–8].

The values of some of these selection criteria can be found easily by querying publicly available bioinformatics resources, including metabolic pathway databases such as KEGG (Kyoto encyclopedia of genes and genomes) [9], protein classification sets such as COGs (clusters of orthologous groups) [10], and databases of “druggable” (potentially useful as drug targets) proteins [5,11,12].

Traditional prioritization approaches such as literature searches and mental integration of multiple criteria can quickly become overwhelming for the researcher. A more effective alternative is computational integration over different criteria to create a ranking function. In this article, we describe such an application—AssessDrugTarget—that ranks the genes in a genome according to a given set of weighted criteria.

### The Need for New Drugs for Tuberculosis

Tuberculosis (TB) is one of the most serious infectious diseases worldwide. The World Health Organization predicts that between 2002 and 2020, 36 million people will have died from TB [13]. Infection occurs via aerosol, and inhalation of only a few droplets containing M. tuberculosis bacilli is sufficient for the pathogen to infect the lungs. Subsequently, the pathogenesis of M. tuberculosis infection occurs in two stages. The first stage, latent TB, is an asymptomatic state that can persist for many years in the host, requiring only a weakened immune response to become activated [14]. In the second stage, active TB, the bacteria begins replicating and causing cough, chest pain, fatigue, and unexplained weight loss. If untreated, the disease eventually culminates in the death of the patient.

The currently available treatment for TB, DOTS (directly observed treatment, short course), lasts for an exhausting 6 mo. The first 2 mo involve a strictly scheduled and monitored intake of four drugs: isoniazid (INH), rifampicin (RIF), pyrazinamide (PZA) and ethambutol (EMB) [15–17]. This phase is followed by a continuation phase of 4 mo of INH and RIF.

#### The problem of persistence.

Only RIF and PZA show activity against “persisters,” organisms that are in the dormant phase. These drugs have helped to substantially shorten the length of DOTS therapy from between 12 and 18 mo to between 9 and 6 mo [16]. However, they do not eliminate all dormant populations, and PZA is likely to affect only those persisters that reside in acidic pH conditions [18].

Another limitation of current TB treatment is that many of the currently used drugs are derived from antibiotics (e.g., RIF and streptomycin), and are cidal only against growing bacterial populations [17].

#### The problem of multidrug resistance.

Even after shortening DOTS to 6 mo, a pertinent practical issue in the treatment is patient compliance; 6 mo is still a lengthy drug administration period and noncompliance most often contributes to the development of multidrug-resistant strains. Alternative second-line drugs then come into use, but multidrug-resistant strains that also exhibit resistance to these second-line drugs are now on the rise [17].

It has also been suggested that targeting already known M. tuberculosis targets for drug development may have limited success because of potential cross-resistance [19]. Thus, new drugs that inhibit new targets and that are difficult to overcome by mutation are required.

### Aims

Here, we present AssessDrugTarget, a new application that aims to rapidly prioritize potential drug targets in a genome, and describe its application to the problem of TB drug development. The need to quickly identify targets that will be effective against persisters (persistence targets) and against growing organisms (new growth targets) has already been highlighted. We propose that by taking advantage of key experiments published on the M. tuberculosis genome, comparative genomic data, and other structured data, scoring schemes can be implemented solely for prioritizing new drug targets in this organism. This approach need not validate all existing and proposed targets, as the criteria for selecting targets depend on the goals of the individual researcher. Since this approach merely prioritizes targets, the subsequent step would be to validate the prioritized targets, for instance by constructing a knockout or using chemical validation. Various “features” are available that can be used to achieve our aims (described below). We use these to prioritize drug targets in TB by the three sets of criteria listed below. In each study, all the available TB features are used but their respective weights are modified to suit the needs of the list.

#### Metabolic drug target criterion.

The top-prioritized target genes must be responsible for unique, growth-essential roles in the TB metabolome (“metabolic chokepoints”). The ranking is further prioritized by lack of homology to the human host and members of the host gut flora, intended to minimize the chances of undesirable host-drug interactions. This approach is expected to bias targets whose metabolic pathways have been mapped.

#### M. tuberculosis-specific drug target criterion.

The prioritized targets must (1) represent growth-essential genes and (2) share close homologs within the Actinobacteria class, but (3) lack a close homolog in the host and host gut flora. Prioritizing by this approach is more likely to yield M. tuberculosis-specific targets, which would be less likely to cross-react with normal bacterial processes in the host. The presence of close homologs in other Actinobacteria will also permit studies of the target in the laboratory. The metabolic pathways of these targets need not be mapped, as in the metabolic drug target criterion.

#### Persistence drug target criterion.

The prioritized targets must play a role in the maintenance of the dormancy phase. This list is not straightforward to produce because persistence is not well understood. We aim to take advantage of the expression profiles of a few targets that have been implicated in maintaining persistence, to evolve the feature weights for this list.

### Data Sources Available for Ranking TB

#### Essential genes.

A novel method, transposon site hybridization, was implemented and applied to determine essential genes in M. tuberculosis under nutrient-rich conditions [8,20]. Briefly, this procedure involved two steps: (1) random disruption of genes by transposon mutagenesis to generate growth mutants, and (2) competitive hybridization between insertion and gene probes to identify mutants that could not survive in nutrient-rich media. This method predicted 614 genes essential for growth in vitro, but with a predicted 1% false discovery rate [8]. Of these predicted essential genes, however, 78% share a close homolog in the degraded Mycobacterium leprae genome (40% the size of M. tuberculosis), which increases confidence in the results on the premise that essential genes will be conserved in the M. leprae genome. Lamichhane et al. [21] used a similar approach to find nonessential genes under the same in vitro conditions: 770 genes were predicted to be dispensable.

These genes have been shown to be essential for survival only under nutrient-rich conditions that favor bacterial growth. The dormancy stage of the macrophage is poorly understood in *M. tuberculosis,* but because the environment in general is harsh, hypoxic, acidic, and nutrient poor, the bacteria maintain a distinctly subdued metabolic state. Therefore, targeting any of these essential genes is more likely to kill or inhibit only growing bacteria unless they are also essential in persisting bacilli.

#### Epidemiology.

In 100 M. tuberculosis clinical isolates, 219 genes were found to be frequently deleted [22]. These genes are undesirable as drug targets, because targeting them would mean patients worldwide would not be treated with the same drug.

#### Druggable protein domains.

Hopkins et al. [23] compiled a database of Interpro domains that bind potent compounds following the Lipinsky rule of 5 (LR5) [11,23]. Briefly, the Investigational Drugs Database, the Pharmaprojects Database, and the literature were each surveyed for LR5-compliant compounds with binding affinity below 10 μM. Only proteins targeted by experimental drugs were retained, and those with activity not modulated by the bound compound were eliminated. The drug-binding domain sequences of these proteins were used to identify the corresponding Interpro domains; 130 protein families were found.

In a similar manner, a database of 70 Enzyme Commission (EC) numbers of known enzyme targets and their respective marketed drugs was compiled [12].

These lists are not specific to M. tuberculosis or even to infectious disease generally, but if the same protein domain has been successfully inhibited in the treatment of another disease, it may help to identify new classes of compounds that are effective against the same target domain in M. tuberculosis.

#### Metabolic chokepoints.

Yeh et al. [24] defined a chokepoint reaction as one that either uniquely consumes a specific substrate or uniquely produces a specific product in the metabolic network. Enzymes involved in unique essential chokepoint reactions would thus make good metabolic drug targets, because the pathway data suggest that their function cannot be compensated for by another enzyme. The number of enzymes having unique EC assignments in the proteome can also be used to indicate which enzymes might perform unique reactions [24].

#### Availability of structural clues.

The availability of a target's crystal structure would aid in rational drug design and would, therefore, provide a strong practical advantage in high-throughput docking and lead optimization studies. Two databases of M. tuberculosis protein crystal structures are available for this purpose, the TB Structural Genomics Consortium [25] and the PDB Database (http://www.rcsb.org/pdb).

The Small Molecule Interaction Database (SMID) genome comparison tool [26] allows the comparison of up to five genomes to identify small molecule–protein domain interactions that are unique to or common among the query genomes. Genome comparisons can be performed to identify possible broad-spectrum targets or pathogen-selective targets. An important point to note here is that the data in the SMID database is based on the analysis of structures in the PDB database that share homology to the query sequence. This means that any results obtained here will be biased by targets that have a close crystallized homolog.

Other structural features such as desirable ranges of length, pI, and molecular mass of the target are also important in the practical tasks of expressing, purifying, and cloning the target.

#### Presence/absence of a close homolog.

An ideal drug would have no or minimal interaction with the host and host flora but high binding specificity for the pathogen. To increase the chances of this favorable binding pattern, targets can be penalized for having high sequence similarity to its host and host flora.

If a broad-spectrum drug or antibiotic is sought, targets can be weighted heavily for having close homolog conserved across a range of pathogens. Conversely, a pathogen-specific target may be sought. A pathogen-specific drug may be desirable for TB, as the long treatment time will encourage selection pressure for drug resistance in the natural gut flora.

#### Gene expression in disease models.

Although the metabolic state of M. tuberculosis in latent in vivo infection is not well understood, various microarray models of the latent state are available [27–33]. If a target is expressed in most of these models, it increases confidence that it is expressed in the latent in vivo infection, which could mean that it is required for survival during dormancy.

## Results

### Growth-Essential Genes

**Figure 1:**
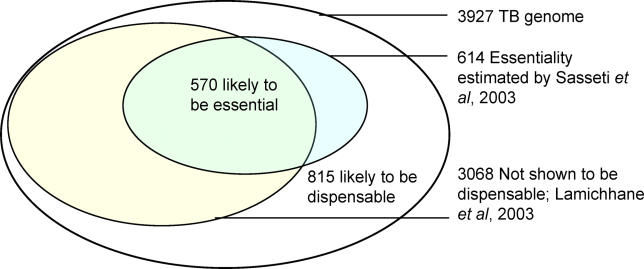
Overlap of M. tuberculosis Growth-Essential Genes

We overlapped the outcome of the two essentiality publications to obtain their consensus (Figure 1). Consolidation of the two lists showed that 570 genes could be essential under nutrient-rich conditions.

### Epidemiology

We found the only two of the 219 deleted genes were in the consolidated essentiality list in the above section. This is a positive finding, because a truly essential gene would be retained in the population.

### Druggable Protein Domains

The M. tuberculosis proteome was scanned for LR5-druggable Interpro domains, and 354 such targets were found. Of these, 50 were members of the combined growth-essentiality list (Table 1).

**Table 1:**
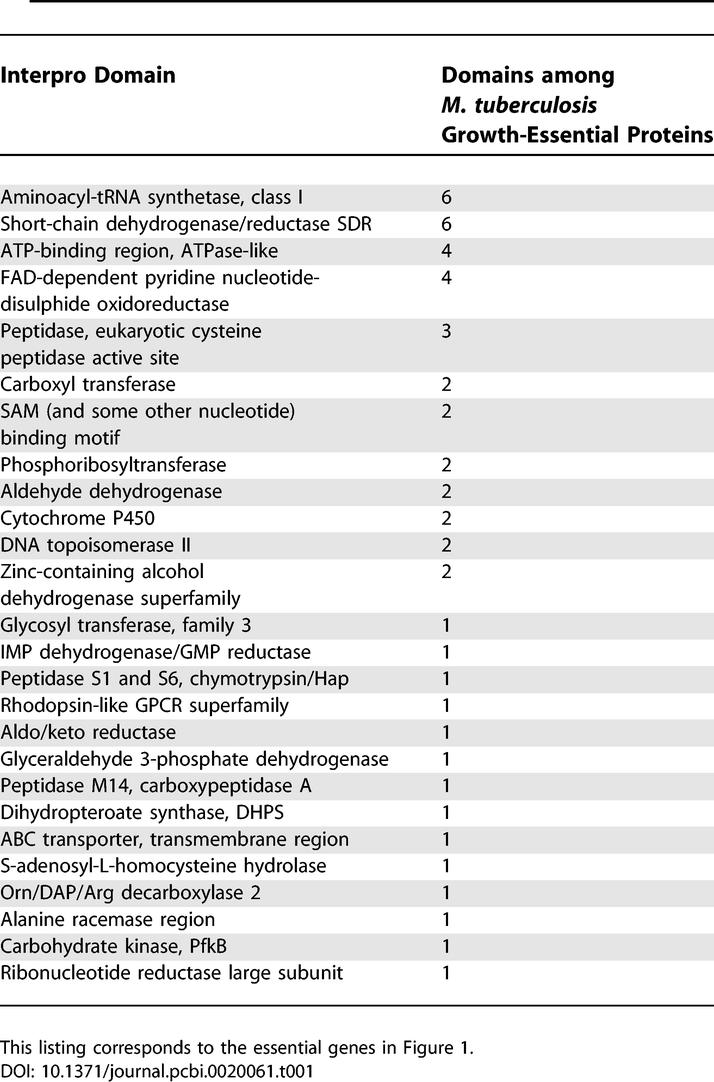
Number of LR5 Compound Binding Domains Predicted in the M. tuberculosis Growth-Essential Proteome

The M. tuberculosis proteome was also queried for the druggable enzyme classes from the Robertson et al. database [12]. Only six growth-essential proteins were found in this search (unpublished data).

### Metabolic Chokepoints

Upon scanning for chokepoint reactions for M. tuberculosis in the KEGG database [9], only 19% of the 3,927-member M. tuberculosis proteome was currently assigned to metabolic pathways. By using chokepoints as criteria for prioritization, the top results become biased to a small fraction of the proteome. This point makes chokepoint analysis quite restrictive. Of the mapped proteins, 51% produce a unique product and 47% consume a unique substrate in the metabolic network. A small fraction (22%) of the TB proteome had been assigned a EC number; of this proportion, 42% was uniquely assigned.

### Availability of Structural Clues

We searched for PDB structures with sequence identity greater than 80% of M. tuberculosis proteins. This threshold was chosen because over 70% sequence identity has been described as useful for drug docking studies [34]. In total, 35 “nutrient-rich” essential targets were found.

We used the SMID genome tool [26] to compare *M. tuberculosis, M. leprae, M. avium, H. sapiens,* and *Mus musculus.* The first three genomes had 102 domains in common that were absent in the latter two.

### Genetic Algorithm-Optimization of Persistence Targets

**Figure 2:**
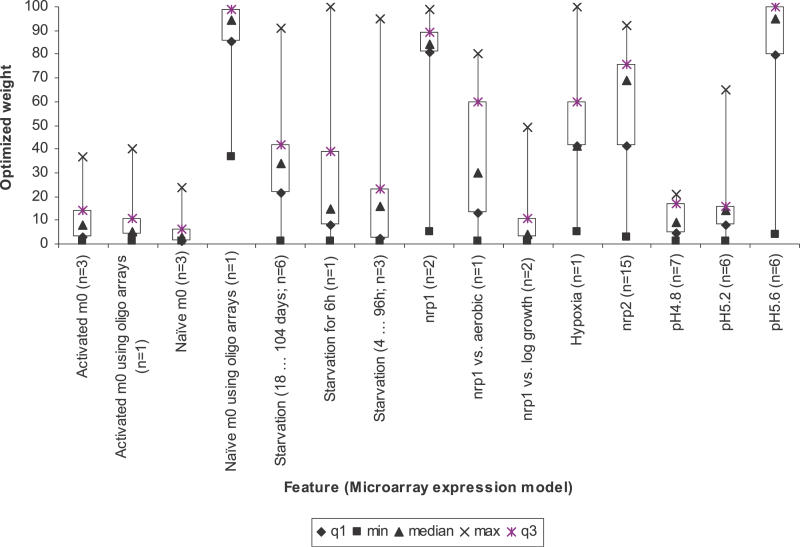
Box and Whisker Plots of GA-Optimized Weights from 100 Evolved Solutions Each possible solution was able to rank eight of ten target genes within the top 25%. M0, macrophage; n, number of experiments; nrp, nonreplicating persistence.

Using Kruskal-Wallis analysis of variance (*p* < 0.0001), the observed mean scores of the optimized weights (Figure 2) had significant within-group differences. The Tukey's HSD (honestly significant difference) test was then applied to all pairwise differences between the means (95% confidence level) to find which groups of experiments evolved the heaviest and lowest weights. This showed that naïve macrophages, in oligo arrays, evolved the heaviest weight (*μ* = 89). This was followed by the grouping of a nonreplicating persistence model, nrp1, and pH 5.6 (μ = 79 and 76 respectively). The lowest evolved weight group included three macrophage-based experiments [27]—activated M0, activated M0 using oligo arrays, and naïve M0—along with a model at pH 4.8 and “nrp1 versus log growth” (μ = 10, 7, 5, 10, and 7, respectively). The medians of 100 possible solutions were used to produce the final optimized list, yielding the ranks seen in Table 2. These optimized weights were able to rank 8/10 targets into the top 25%.

**Table 2:**
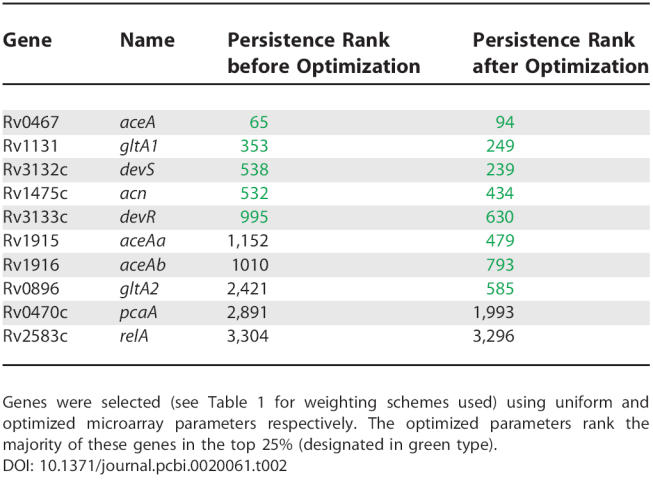
Ranking of Persistence-Implied Genes with Only Latent-State Model Features

## Discussion

### A Quick and Flexible Decision-Making Tool

Using AssessDrugTarget to prioritize drug targets is very quick, taking a few seconds to produce a desirable list. The critical part of using the software is to carefully choose which features to use and how much to weight them so that the prioritization demands set out by the researcher are met as closely as possible. These decisions should be influenced by the reliability of the respective datasets. Alternatively, if a set of example targets is available, an optimization technique such as a genetic algorithm (GA) can be used to determine weights.

Here, we performed three studies on M. tuberculosis to produce metabolic, M. tuberculosis-specific, and persistence target ranks for all members of the genome (Dataset S1). We do not discuss new targets here because we wish to focus on the ability to identify new drug targets and not on the subsequent stage of drug discovery, target validation [2]. Therefore, we assess how current, candidate, and proposed M. tuberculosis targets (hereafter “studied targets”) stand in our rankings (Table 3) and use the observations to evaluate the strengths and limitations of software-based target prioritization and to explore possible improvements to the approach. The three classes of studied targets (Table S1) were obtained from a literature review. In Table 3, we use a crude “top 13%” threshold (representing ranks >500) to draw attention to genes that were prioritized.

**Table 3:**
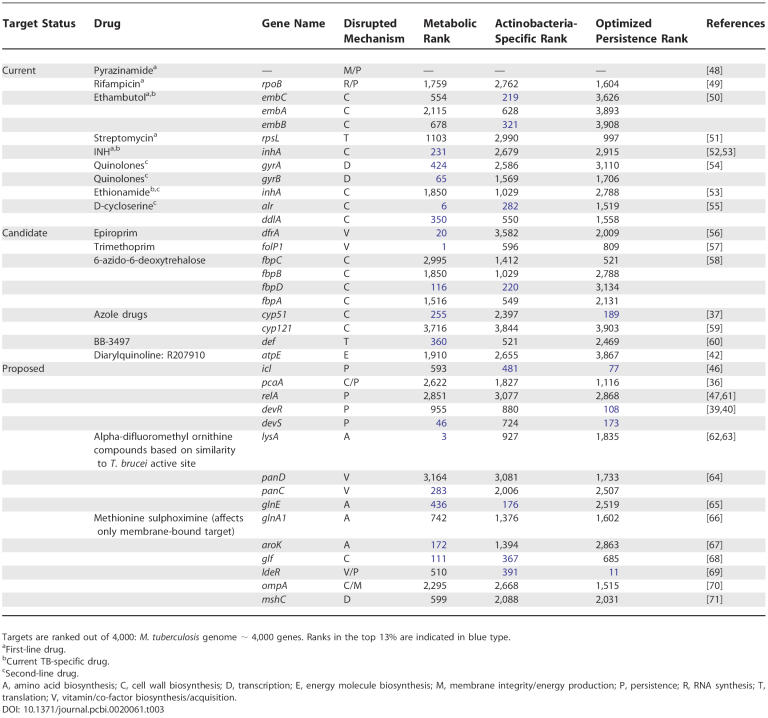
Ranks of Studied Targets in Three Prioritized Lists

### Functional Category Biases of Studied TB Drug Targets

Table 3 (target status: current and candidate) shows that 60% of current and candidate targets are involved in cell wall biosynthesis. It has been noted, however, that drugs that target cell wall synthesis are more likely to be active against growing bacteria than against persisters [35]. Also, both RIF and PZA, which are the only drugs to shorten TB chemotherapy (and have thus shown activity against persisters), do not target cell wall biosynthesis. Therefore, new targets—to combat persistence—are required, and this explains why 30% of newly proposed targets belong to this category (Table 3, target status: proposed). Only two of the proposed targets *(pcaA, glf)* are involved in cell wall biosynthesis, although *pcaA* is a more interesting target for its role in persistence [36].

Of course, it would be desirable if the target selected is essential for both growing and dormant bacteria. Such targets were expected to have high ranks in both the metabolic and optimized persistence lists. It was seen that AssessDrugTarget ranked two of the studied targets, *cyp51* and *devS,* in the top 13% of both lists (Table 3). These are discussed later.

### Ranking of Studied Targets

#### Metabolic list.

It was firstly observed that most of the targets, inhibited by current second-line TB drugs, could be prioritized into the top 13% of the metabolic list (Table 3). These are involved in cell wall synthesis or transcription. Of the first-line drug targets, only *inhA* could be prioritized within the top 13% (rank = 231). Of the three targets of the first-line drug EMB, two rank fairly highly: 554 *(embC)* and 678 *(embB)*. These two targets, and not *embA,* were shown by transposon site hybridization mutagenesis to be essential, leading to their ranks being vastly prioritized over *embA* (rank = 2,115). Because we were interested in targets that carry out unique reactions, the *embC, A,* and *B* genes generally rank lower in the metabolic list because all three catalyze the same reaction. Similarly, four RNA polymerases in *M. tuberculosis (rpoA, B, C,* and *Z)* reduce the rank of the SM polymerase target *rpoB* (rank = 1,759).

The top three targets in this list have each been studied for drug discovery: *folP1* (rank = 1), *lysA* (rank = 3), and *alr* (rank = 6). The *def* gene, coding for peptide deformylase, is a new target for the treatment of multidrug-resistant *M. tuberculosis;* it also ranks highly in this list (rank = 360).

Other functional classes that were represented in the prioritized target list include vitamin and amino acid biosynthesis. Two members of the top list (*folP1* and *dfrA,* ranked at 1 and 20, respectively) are involved in the biosynthesis of folate, an essential vitamin in *M. tuberculosis,* indicating this to be an important pathway to target for drug development.

#### Actinobacteria-specific list.

Of the three currently used drugs that have been shown to specifically inhibit TB (Table 3), INH and ETA both target *inhA* and EMB targets *embC, A,* and *B*. Only EMB targets could be prioritized as being M. tuberculosis-specific (Table 3): *embC* and *B* rank higher than our 13% criterion, and *embA* ranks 628. The failure to prioritize *inhA* (rank = 2,679) suggests that subtle structural features, rather than sequence homology, can be used to enhance ranking it as a M. tuberculosis-specific target. These could involve Pfam scores.

One of the fibronectin-binding proteins, *fbpD,* also rank highly in the M. tuberculosis-specific list (rank >13%) (Table 3). Two persistence targets may also be uniquely targeted in *M. tuberculosis: icl* (rank >13%) and *devS* (rank = 724).

The range of top ranks of the studied targets are not as high as in the metabolic list; the top rank is 176 *(glnE)* compared to three targets ranked above 10 in the metabolic list. This is somewhat expected because the pathways of many known targets are mapped and, therefore, are likely to be represented in the metabolic list. It does suggest, however, that many new M. tuberculosis-specific targets can be found.

#### Persistence list.

Out of the seven persistence targets shown in Table 3, *icl, devR,* and *devS* were already optimized to rank in the top 25% (Table 2); *pcaA* and *relA* could not be optimized. he target *devS* also has a high metabolic rank. As mentioned earlier, *cyp51,* one of 20 M. tuberculosis cytochrome P450s, ranked highly both in this list and in the metabolic list. It has been shown that niclosamide (antihelminth group) and 2-nitroimidazole (antifungal group), which are substrates of cytochrome P450s, exhibit activity against stationary phase M. tuberculosis [37] so they may inhibit *cyp51* in this process. *IdeR,* an iron-dependent repressor and activator, was the top-ranking persistence target from this list (rank = 11) and it has been shown to be involved in oxidative stress response [38].

### Observed Values of Individual Features

Individual feature scores not only contribute to the overall target ranks but also embody the properties of a ranked target. Some features may be more useful than others in the context of M. tuberculosis targets. Table 4 lists the properties observed for the studied targets. The contributions of individual features to the ranking of these targets is discussed next.

**Table 4:**
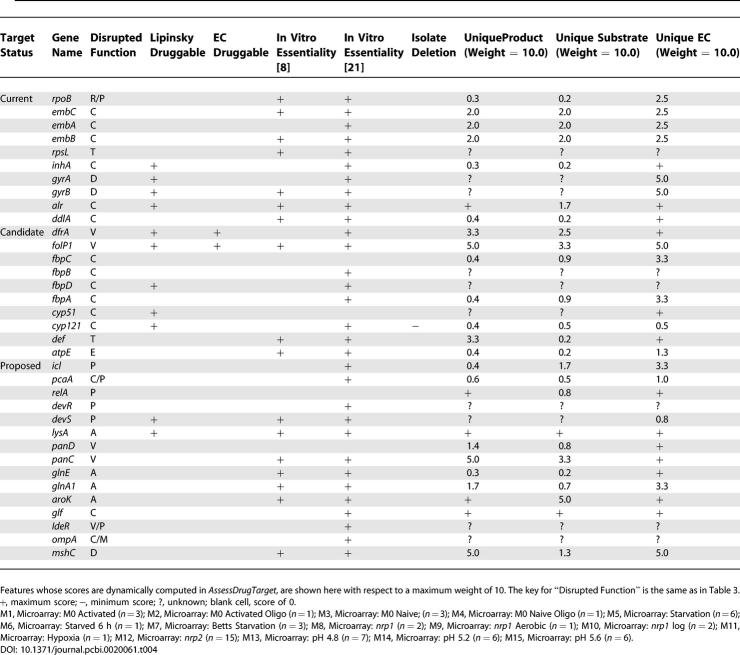
Individual Features of Studied Targets

**Table 4:**
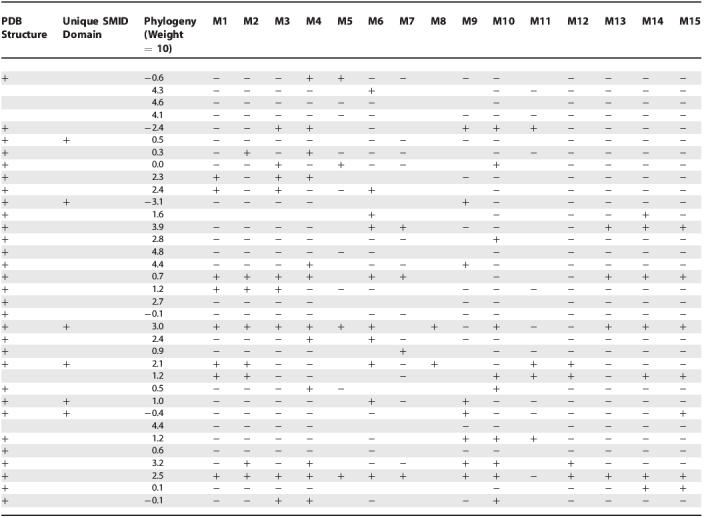
Extended

#### Druggability.

It was mentioned earlier that 50 LR5-compliant targets are found among the growth-essential M. tuberculosis genes (Table 1). Among the 35 studied targets, 30% appear to bind a LR5-compound (Table 4) and only two could be identified as potential targets from the druggable enzyme database [12]. However, these two targets overlap the LR5 predictions.

The only potential studied target that did not have an inhibitor listed in Table 3 but that could possibly bind a LR5-compliant compound, was the two-component sensor histidine kinase *devS* [39,40], a potential persistence target. *devS* has an ATPase domain that is structurally similar between histidine kinases and DNA gyrases (for the domain description, see http://www.sanger.ac.uk/cgi-bin/Pfam/getacc?PF02518). Coumarins are a class of LR5 drugs that competitively bind to the ATPase domain of DNA gyrases and are used in the treatment of human cancer (http://www.embl-ebi.ac.uk/interpro/DisplayIproEntry?ac=IPR000565). This class of compounds could be studied for activity against *devS*.

#### Growth-essentiality and epidemiology.

In one study [8], 16 of 34 (47%) studied targets were found to be essential for growth in vitro. In another study [21], 31 of 34 (91%) studied targets were not shown to be dispensable under similar growth conditions (Table 4). Since only half of the studied active-M. tuberculosis targets were identified in the first study, it is likely that more growth targets can be found. Coincidentally, the percentages of studied targets classed as essential (47% and 91% respectively) are proportional to the estimated percentages of essential genes in the M. tuberculosis genome: 15% and 35% in the two respective studies [8,21]. *devS*, a potential persistence target, appeared in both lists, suggesting that inhibiting it might affect growing as well as dormant M. tuberculosis.

The only studied target that was found to be deleted in clinical isolates was the cytochrome P450 oxidase *cyp121* [22]. This would still be a good target for intervention because M. tuberculosis contains 19 other P450 oxidases that are likely to be sensitive to treatment by azole drugs. *icl2,* a second homologous copy of the persistence target *icl1,* was also found to be deleted in the same study. Once again, this is acceptable given that M. tuberculosis has two copies of this gene and can presumably tolerate the loss of one of them.

#### Metabolic chokepoints.

In total, 29 (77%) of the studied targets are mapped to metabolic pathways (Table 4). Of these, only 15% targets produce a unique product and 7% consume a unique substrate. The unique product and unique substrate criteria of identifying chokepoints, therefore, has limited representation in studied targets. It should perhaps be noted that the perceived “uniqueness” of many of these predicted chokepoint reactions may be negated by the presence of alternative and yet uncharacterized pathways in M. tuberculosis. Since only 19% of the M. tuberculosis proteome has been mapped to metabolic pathways, the unmapped 81% represents a relatively large unknown fraction that can delimit the value of chokepoint prediction in M. tuberculosis. Only further laboratory knockout studies (for example, by demonstrating that a lethal auxotrophic mutant can be generated) will show how many of these chokepoint reactions really are unique in M. tuberculosis.

An alternative application of pathway data for drug target prioritization might be to characterize the downstream essential steps, in the same and subsequently linked pathways, that are disrupted by knocking out an early point in the pathway.

We also sought the number of enzymes with a unique EC assignment, assuming that it would indicate the number of chokepoint targets. This category contains 17 (50%) of the studied targets (Table 4). Therefore, this indicator may be more predictive of chokepoint reactions in M. tuberculosis than the unique product and unique substrate indicators.

#### Structural clues.

Some 85% of the studied targets have been crystallized, highlighting the important contribution to TB drug discovery studies by the M. tuberculosis structural genomics consortium and other structural genomics groups (Table 4) [25]. Of the studied targets in Table 4,6 (20%) were predicted to have small molecule interaction domains (SMID) present in mycobacteria but absent in human and mouse (Table 4). One of these was *inhA,* which ranked quite low in the M. tuberculosis-specific list (Table 3; rank = 1,029). The gene *inhA* has 25% sequence identity to a gene encoding a human protein *(pecR)* and 31% to a mouse protein *(Decr1)*. Thus, although *inhA* ranked quite low when penalized by host sequence identity, the SMID domain feature scores it positively as a “host-safe” target. However, SMID predicted that inhA would interact with triclosan (http://www.quantexlabs.com/triclosan.htm), a broad-spectrum antibacterial/antimicrobial agent. Therefore, the presence of *inhA* in host flora (e.g., 33% identity to *E. coli FabI*) would still have rendered this target a low rank in M. tuberculosis-specific prioritization. This does suggest, however, that prioritization of *inhA* can be achieved by selecting a heavy weight for the SMID domain feature and other subtle structure-based features. In the future, we plan to use the Pfam scores [41] when sequence similarity of the target to the host and host flora is very high.

#### Expression in latent state models.

As mentioned earlier, genetic programming could optimize the weights of latent-state microarray models to prioritize 80% of genes known to be involved in persistence. This programming approach helped us to create a persistence-optimized list. Scanning the top ranks of the optimized persistence list thus presents a chance to find more targets that could be LR5-compliant and that could help to target persistence (altogether, 354 LR5-compliant targets were found in M. tuberculosis).

### Studied Targets That Were Not Prioritized

We were unable to prioritize 25% of studied targets in any of our three lists (Table 3). Certain targets, such as ATP synthase, encoded by *atpE* (ranks of 1,910, 2,655, and 3,867, respectively), do not fit any of the criteria we sought. ATPE is a ubiquitous protein required by all organisms, so it would not have been a logical choice as a drug target. The actual discovery of the target was itself quite a surprise [42], so it is unlikely that it would be prioritized in our computational approach. The lesson is that some targets are still best discovered serendipitously.

### Future Development

Researchers may wish to extend the use of our software to dissect the prioritized list by desirable ranges of peptide length, pI, and molecular mass if they wish to express, purify, and clone a desired target. We plan to incorporate the Pfam scores for selective targeting when the sequence homology to a host protein is high. Certain structured data that are not yet possible to predict computationally, but that would boost the software-based approach, include pathway-specific auxotrophic mutant data and putative transporters of essential molecules.

### Conclusion

AssessDrugTarget provides a simple framework for integrating the vast amount of biological data that can be used in the drug target identification stage. The software can be extended to include scoring patterns for any kind of structured biological data. The weights given to each criterion can be set by an expert user or determined using a GA if example targets are available. We were able to predict 354 LR5-compliant protein domains in M. tuberculosis. One of the two growth-essentiality datasets correctly identified 90% of studied active-TB targets, whereas the other was 55% accurate in this respect. We found that the unique product and unique substrate criteria of chokepoint analysis may be of limited value because of the relatively small proportion of M. tuberculosis proteins that have been mapped to metabolic pathways (19%). The chokepoint criterion of unique EC assignments may be more useful for predicting M. tuberculosis targets. Predicting distinct SMID domains and using other structure-based features, such as Pfam scores, may be useful for prioritizing targets when sequence homology to a host protein is relatively high. The various microarray models of latent TB allow us to prioritize which targets could be selected for combating TB persistence.

## Materials and Methods

Our application, AssessDrugTarget, accepts a parameter file in XML format. The XML file specifies a number of desired drug target features, each of which has a user-specified weight, penalty scores, and at least one associated SQL query. A null score must also be specified if the feature data are not available for a particular gene. The software generates a table in which the genes are ranked by their score totaled across the provided feature weights. Optional information, for example the gene's alternative names, its mapped metabolic pathways, and references, will also be printed if these are specified under the “supplementary” tag of the parameter XML file.

### Implementation.

AssessDrugTarget is written in Perl and has been designed to be as flexible and extendable as possible. It comes bundled with a DrugTarget package and requires the following additional Perl modules, which are all available from the CPAN repository (http://www.cpan.org): XML::TreeBuilder, DBI, and Statistics::Descriptive.

The parameter XML file initially specifies a database resource that contains information about the database type and its connection attributes. The Perl database interface (DBI; http://dbi.perl.org) was used to implement this feature. DBI allows the XML file to be modified to connect to different kinds of SQL databases using any valid Perl DBI driver. The SQL query for each drug target feature is specified in the main parameter file. We plan to expand this capacity in the future to receive XML responses from a web service and process them to generate DrugTarget::Report objects.

The scores and penalties in the parameter XML file can be modified easily to bias the weights according to the confidence the researcher has in a particular drug feature type.

To specify new drug features, new objects representing the feature can be created by (1) extending the DrugTarget::ReportGenerator object; (2) implementing it to return a DrugTarget::Report object (an object in which each gene must be scored); and (3) specifying it as a valid feature in the DrugTarget::ReportParser class data and creating an entry in the XML parameters.

In our target genome, M. tuberculosis strain H37Rv, each gene has a unique “Rv” code number assigned that was the synonym used to index all tables in our database. All the original datasets we used could be mapped back to this code. Such a numeric indexing system should always be preferred over using gene names or other qualitative variables, which often change upon data revision. In addition, results of BLAST searches [43] were also indexed by this code. Other ways of indexing genes that could be used toward this end could involve mapping them back to their respective orthologous clusters [44].

### Weight selection for metabolic and M. tuberculosis-specific target lists.

The weights for the different features used to achieve the first two ranked lists are summarized in Table 5. The weights were chosen in consultation with a TB drug development group.

**Table 5:**
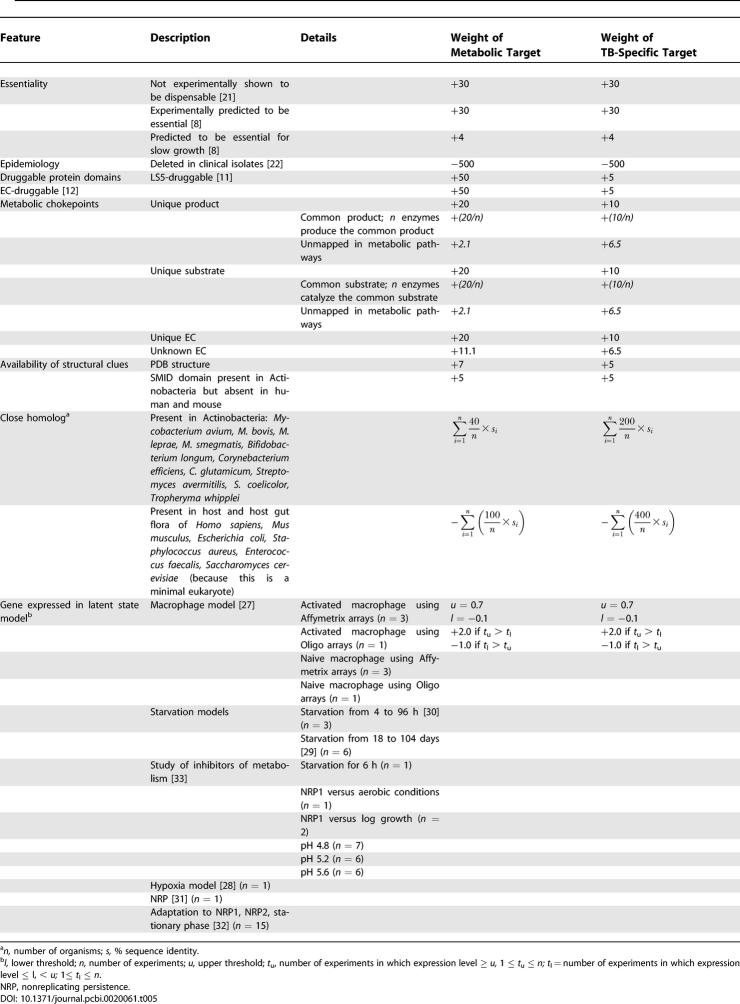
Weighting Schemes of Different Features Used to Prioritize TB Drug Targets

### Growth-essentiality and epidemiology.

Both growth-essentiality and epidemiology were weighted heavily in both the metabolic and M. tuberculosis-specific lists (+30 if essential and −500 if deleted, respectively) (Table 5), because it was considered vital in our study that the prioritized targets play essential roles and be conserved across clinical strains.

### Druggable protein domains.

A heavy weight, +50, was chosen for the “druggable” domain feature in the metabolic list (Table 5). We chose this weight because we expected the chances of finding a known “druggable” domain to be much higher in proteins, which could be mapped to known metabolic pathway(s). In the M. tuberculosis-specific list, many of the prioritized targets were expected to be classed as “conserved hypothetical proteins” with unknown function, and were therefore unlikely to possess a known “druggable” domain. Consequently, a low weight, +5, was assigned to this feature in the M. tuberculosis-specific list.

### Metabolic chokepoints.

We wished the metabolic chokepoint feature to dominate the metabolic list because we wanted to prioritize targets that carry out unique metabolic roles. Consequently, it was assigned a heavy weight, +20, in the metabolic list (Table 5). For the M. tuberculosis-specific list, the same feature was assigned a lower weighting of +10, because we wanted the scores of the “close homolog” feature to dictate the rankings in this case.

### Availability of structural clues.

The weights for these features were kept quite low (<7; Table 5) for both lists because these confer mainly pragmatic advantages, which would not be the primary consideration in identifying a new drug target. Often in a drug discovery program, the target is cocrystallized with the lead compound at a latter stage, regardless of whether a crystal structure was available at the start or not.

### Presence or absence of a close homolog.

One of our goals was to prioritize M. tuberculosis-specific targets. Therefore, positive weights were assigned to targets with close homologs conserved across the Actinomycetes group in order to minimize interactions with any other bacterial group (no other Actinomycetes members are known to symbiotically reside within human tissue). Negative weights were assigned if the target was found in the host or in gut flora (Table 5). Homology to gut flora was penalized because the treatment for TB is still quite lengthy, and undesirable interactions with symbiotic bacteria may not be well tolerated by the patient.

We wanted this feature to dominate the M. tuberculosis-specific list but not the metabolic list. Correspondingly, the weights and penalties chosen were of much higher magnitude in the M. tuberculosis-specific study (+200 and −400, respectively) than in the metabolic list (+40 and −100) (Table 5).

### Gene expression in disease models.

Each microarray experiment was first normalized to have a mean intensity of 0.0 and a variation of 1.0 in order to remove some of the inherent effects of variation associated with microarray technology [45]. To be available for a drug-target interaction, the target needs only to be present: It does not require expression levels many times higher than its reference state. For this reason, a relatively low upper expression threshold (>0.7) was chosen to indicate detectable expression in a microarray experiment (Table 5). A lower expression threshold of less than −0.1 was chosen to penalize targets whose expression may be difficult to detect.

This feature was given much lower weighting than any of the other features (weights and penalties of +2.0 and −1.0, respectively) because of the high level of variability in reproducing microarray results. We also did not have access to the underlying raw data for a number of these experiments, so the low weights also play a part in balancing this irregularity. The actual fold change in expression does not affect the results significantly, as the weighting takes into account only expression levels below or above user-specified thresholds.

### GA implementation for optimizing persistence targets.

The microarray expression models of latent state (Table 5) help identify the genes that might be required for persistence. In order to produce a prioritized persistence target list, we therefore wanted to produce a list in which (1) these expression features were weighted heavily with respect to the remaining features, and (2) the respective set of weighted expression features prioritized genes currently shown to be involved in maintaining M. tuberculosis persistence. The relative importance of each model to persistence was not known, so a GA was implemented (the GA package used is part of the Biojava toolkit, available at: http://www.biojava.org) to evolve the weights, which would satisfy these two requirements.

The GA fitness function was designed to evolve a set of weights that prioritized genes involved in persistence [36,39,46,47] above a specified threshold (Table 2). We chose a threshold of 1,000, which represents the top 25% of the M. tuberculosis genome (lower thresholds were tried but weights could not be evolved that would rank all the proposed persistence targets above these thresholds). The algorithm was given a population of weights ranging from 1 to 100 to mutate and cross over from, yielding an optimized set of weights (Figure 2).

In terms of performance, this algorithm is expected to scale linearly with the number of genes. The complexity of the scoring system will also affect performance. In our approach, we cached the scores for a prior set of weights, and these were scaled every time the algorithm assigned new weights. We had only ten known persistence targets that we could use for training; ideally, a much larger training set (>100) would have been preferred.

## Supporting Information

Dataset S1 The Ranks of M. tuberculosis Genes (A) Metabolic, (B) M. tuberculosis-specific, and (C) persistence drug targets are presented in an Excel spreadsheet.
(1.3 MB XLS)

Table S1 Accession and ID Numbers of Possible M. tuberculosis Target Genes and Proteins (31 KB PDF)

### Accession Numbers

The GenBank (http://www.ncbi.nlm.nih.gov) accession numbers of the genes referred to in the text are *Decr1* (NP_080448) and *pecR* (NP_060911); and for M. tuberculosis strain H37Rv (AL123456).

## Abbreviations

DOTS: directly observed treatment, short course
EC: Enzyme Commission
EMB: ethambutol
GA: genetic algorithm
INH: isoniazid
LR5: Lipinski rule of 5
PZA: pyrazinamide
RIF: rifampicin
TB: tuberculosis
