# Supplementary material for: Prioritizing Genomic Drug Targets in Pathogens: Application to Mycobacterium tuberculosis
Source: PLoS Comput Biol. 2006 Jun 9;2(6):e61. doi: 10.1371/journal.pcbi.0020061 (PMC1475714; doi:10.1371/journal.pcbi.0020061)
Supplement: Supplementary file 1 [file pcbi.0020061.st001.pdf]

locus_tag	Gene name	Synonyms	NCBI_nucleotide_sequence	Entrez_gene_ID	Refseq_protein_accession_ID	SWISS-PROT
Rv0005	gyrB	Rv0005	15607147	887081	NP_214519	P41514
Rv0006	gyrA	Rv0006	15607148	887105	NP_214520	Q07702
Rv0129c	fbpC	85C, fbpC2, mpt45, Rv0129c	57116693	886885	YP_177694	P31953
Rv0429c	def	Rv0429c	15607570	886366	NP_214943	P96275
Rv0467	icl	aceA, Rv0467	57116734	886291	YP_177728	O53752
Rv0470c	pcaA	Rv0470c, umaA2	57116736	886284	YP_177730	Q7D9R5
Rv0667	rpoB	Rv0667	15607807	888164	NP_215181	Q9XC84
Rv0682	rpsL	Rv0682	15607822	888259	NP_215196	P41196
Rv0764c	cyp51	Rv0764c	15607904	888819	NP_215278	P77901
Rv0899	ompA	Rv0899	15608039	885286	NP_215414	Q10557
Rv1267c	embR	Rv1267c	15608407	887026	NP_215783	Q11052
Rv1293	lysA	Rv1293	15608433	886960	NP_215809	P31848
Rv1305	atpE	Rv1305	15608445	886937	NP_215821	Q10598
Rv1484	inhA	Rv1484	15608622	886523	NP_216000	P46533
Rv1886c	fbpB	85B, mpt59, Rv1886c	15609023	885785	NP_216402	P31952
Rv2031c	hspX	acr, Rv2031c	15609168	887579	NP_216547	P30223
Rv2130c	mshC	cysS2, Rv2130c	15609267	887492	NP_216646	O33264
Rv2220	glnA1	glnA, Rv2220	15609357	888383	NP_216736	Q10377
Rv2221c	glnE	Rv2221c	15609358	887995	NP_216737	Q10379
Rv2276	cyp121	Rv2276	15609413	888373	NP_216792	Q59571
Rv2539c	aroK	Rv2539c	15609676	887434	NP_217055	P95014
Rv2583c	relA	Rv2583c	15609720	887888	NP_217099	Q50638
Rv2711	ideR	dtxR, Rv2711	15609848	888590	NP_217227	Q50495
Rv2763c	dfrA	folA, Rv2763c	15609900	887777	NP_217279	O33305
Rv2981c	ddlA	ddl, Rv2981c	15610118	888415	NP_217497	P95114
Rv3132c	devS	Rv3132c	15610268	888829	NP_217648	P95194I
Rv3133c	devR	dosR, Rv3133c	15610269	888842	NP_217649	P95193I
Rv3134c	Rv3134c		15610270	887558	NP_217650	P95192I
Rv3423c	alr	Rv3423c	15610559	887634	NP_217940	Q50705
Rv3601c	panD	Rv3601c	15610737	885596	NP_218118	O06281
Rv3602c	panC	Rv3602c	15610738	885459	NP_218119	O06280
Rv3608c	folP1	Rv3608c	57117134	885831	YP_177997	O06274
Rv3793	embC	Rv3793	15610929	886112	NP_218310	P72059
Rv3794	embA	Rv3794	15610930	886123	NP_218311	P72060
Rv3795	embB	Rv3795	15610931	886126	NP_218312	P72030
Rv3803c	fbpD	fbpC1, mpb51, mpt51, Rv3803c	57117159	886121	YP_178017	Q48923

Rv3804c	fbpA	85A, mpt44, Rv3804c	15610940	886132	NP_218321	P17944
Rv3809c	glf	ceoA, Rv3809c	15610945	886142	NP_218326	O06934
